# Our Sleeping Rooms

**Published:** 1886-03

**Authors:** 


### OUR SLEEPING ROOMS.'

It is to be regretted that paperings or carpetings should ever be used in the sleeping room. Alas! what evil is lurking in the area of the four square walls which encompass us! What enemy is that, although trodden upon, yet is not subdued! Let the walls of our sleeping rooms be kalsomined and the carpets removed from the floors. Let the crevices be carefully filled with putty (anyone can do this) and the floor neatly painted or stained. A rug at the bedside, with small ones at the bureau and commode (Kensington rugs), will relieve the nakedness of the floor. These should be carried out weekly, thorougbly shaken, and exposed for an hour to sun and wind. Towels and wash-cloths used during the day should never remain in the room during the night. I have seen wash-cloths, used day after day in a sleeping room, become sour and musty, emitting a strong odor both disagreeable and unhealthy. The water-can and the entire toilet-set must be kept perfectly sweet and pure. I do not mean merely clean to the eye, but clean enough for the chemist's use. Attention must also be called to the tooth-brush, which should always be thoroughly cleaned after using, and placed, handle down, in an upright holder. I have found odor enough about one tooth-brush to infect the atmosphere of a common sleeping room. In regard to ventilation, open as many doors and windows as permissible, avoiding a draft; but moving air is indispensible to the health of the sleeper. Let the bed stand as near the center of the room as possible, but on no account close to the wall. No one housekeeper may be able to carry out all these suggestions, but it is the ideal, or housekeeping as it ought to be, which should be held up to the eye of the reader, that each one may choose what she can best carry out in her daily practice.—*Good Housekeeping.*

